# Genomic, Probiotic, and Functional Properties of *Bacteroides dorei* RX2020 Isolated from Gut Microbiota

**DOI:** 10.3390/nu17061066

**Published:** 2025-03-18

**Authors:** Siqin He, Liqiong Song, Yuchun Xiao, Yuanming Huang, Zhihong Ren

**Affiliations:** National Key Laboratory of Intelligent Tracking and Forecasting for Infectious Diseases, National Institute for Communicable Disease Control and Prevention, Chinese Center for Disease Control and Prevention, Beijing 102206, China; hesiqin97@foxmail.com (S.H.);

**Keywords:** *Bacteroides*, probiotic properties, next-generation probiotics, bioactive metabolites

## Abstract

Background/Objectives: Gut microbiota is essential for maintaining host immune homeostasis and has been confirmed to be closely related to some intestinal and extraintestinal diseases. *Bacteroides*, as the dominant bacterial genus in the human gut, has attracted great attention due to its excellent metabolic activity, but there are few studies on *Bacteroides dorei* species. In our previous study, a gut commensal strain, *Bacteroides dorei* RX2020 (*B. dorei*), was isolated from healthy human feces and exhibited superior flavonoid metabolic activity, prompting further analysis of its uncharacterized genomic features, probiotic potential, safety, and immunomodulatory activity. Results: The results showed that *B. dorei* exhibited intrinsic probiotic functionalities with preserved genomic and phenotypic stability, demonstrated safety profiles in murine models through in vivo assessments, and conferred antagonistic activity against enteric foodborne pathogens via competitive exclusion. The strain also demonstrated abundant metabolic activity and was involved in the metabolism of tryptophan and bile acids (BAs). Moreover, *B. dorei* can promote the production of IFNβ by dendritic cells (DCs) to inhibit the replication of influenza virus in epithelial cells, which may be achieved by regulating host metabolism. Conclusions: This study reveals the potential of *B. dorei* as next-generation probiotics (NGPs), contributing to a broader understanding and application of these novel probiotics in health and disease management.

## 1. Introduction

Traditional probiotics, predominantly *Lactobacillus* and *Bifidobacterium* strains, have been extensively utilized in food and dietary supplements due to their well-documented roles in gut microbiota modulation, digestive enhancement, and immune regulation [1]. The rapid development of next-generation sequencing (NGS) technologies has significantly advanced our understanding of the compositional dynamics and functional potential of the human gut microbiome. The first decade of gut microbiota research focused on DNA-based 16S rRNA gene sequencing and shotgun metagenomic sequencing, elucidating microbial composition and gene content. The addition of high-throughput sequencing technology further revealed genetic diversity and molecular ecological information such as species composition, gene function, and metabolic pathways [2]. This technological breakthrough has not only deepened our insights into microbial ecology but also facilitated the identification and characterization of novel microbial taxa [3]. Consequently, the conceptual framework of NGPs has been progressively established. NGP refers to live microbial therapeutics developed from a diverse range of microbial populations, primarily aimed at treating specific diseases rather than traditional health maintenance. These probiotics transcend the species limitations of conventional probiotics, representing a shift from dietary supplements to targeted medical products [4].

Currently, three prominent NGPs—*Akkermansia*, *Bacteroides*, and *Clostridium*—have attracted research interest in their therapeutic potential and clinical applications. The abundance of *Akkermansia muciniphila* (*A. muciniphila*) was significantly reduced in T2D, and supplementation of *A. muciniphila* alone is sufficient to protect mice against high glucose-induced glucose intolerance and has a positive effect on the pathogenesis of T2D and obesity [5,6]. A systematic investigation of gut microbial profiles across 3409 human subjects revealed Bacteroidetes had the strongest correlation with the diversity of the gut microbiota [7]. *Bacteroides* can promote host health by regulating gut immune homeostasis, resisting the colonization of pathogenic bacteria, inhibiting inflammatory response, regulating obesity-induced glucose intolerance, and inducing the proliferation of probiotic bacteria, among other means. For example, *Bacteroides acidifaciens* enhances colonic IgA synthesis, supporting intestinal mucosal integrity through selective clearance of epithelial-invading pathogens [8]. *Bacteroides uniformis* supplementation can alleviate DSS-induced colitis by regulating Treg/Th17 balance [9]. In addition, an inverse correlation between *B. dorei* abundance and SARS-CoV-2 viral load was observed in feces samples from COVID-19 patients [10]. *Clostridium orbiscindens* exerts anti-influenza effects through flavonoid-derived deaminotyrosine (DAT) production, which enhances type I interferon (IFN-I) signaling [11].

In our previous study, we conducted a comprehensive screening of 48 bacterial strains isolated from fermented foods and healthy human feces to evaluate their metabolic capacity for the flavonoid quercetin [12]. Among these, a *B. dorei* strain derived from healthy human feces demonstrated optimal metabolic capacity, leading to its selection for further characterization. However, the genomic characterization, probiotic properties, and safety profile of *B. dorei* remain largely unexplored. In this study, we performed comprehensive whole-genome annotation of *B. dorei* using multiple databases and systematically evaluated its potential as a probiotic candidate through an integrated analysis of phenotypic traits and genetic determinants. This included assessments of general characteristics, genetic stability, antibiotic resistance, tolerance to simulated gastrointestinal conditions, in vivo toxicity, and the expression of functionally relevant genes. Furthermore, the metabolic and immunomodulatory activity of *B. dorei* was investigated to elucidate the molecular mechanisms underlying its probiotic properties and mechanisms of interaction with the host, thereby addressing critical knowledge gaps in its functional characterization.

## 2. Materials and Methods

### 2.1. Bacterial and Animals

*Bacteroides dorei* RX2020 was previously isolated from the feces of a healthy female adult volunteer in Beijing, China by our group (CGMCC No. 21251), and cultured in brain heart infusion (BHI) agar (Thermo Fisher Scientific, Waltham, MA, USA) supplemented with 5% sterile defibrinated sheep blood or Anaerobe Basal Broth (Thermo Scientific™ Oxoid, Basingstoke, UK) at 37 °C for 24–48 h under anaerobic conditions.

*Lactobacillus plantarum* DOMLa (*L. plantarum*), *Staphylococcus aureus* B220-7 (*S. aureus*), *Escherichia coli* EDL933 (EHEC) (*E. coli*), *Salmonella typhimurium* 1344 (*S. typhimurium*), and *Enterococcus faecalis* ATCC51299 (*E. faecalis*) were the early storage of our group. *L. plantarum* was cultured overnight in MRS broth (Thermo Scientific™ Oxoid, Basingstoke, UK) at 37 °C, while *E. coli*, *S. aureus*, *S. typhimurium*, and *E. faecalis* were streaked onto LB agar plates (Thermo Scientific™ Oxoid, Basingstoke, UK) and incubated overnight at 37 °C in a 5% CO_2_ atmosphere.

Influenza A virus A/Puerto Rico/8/34 mouse lung-adaptive strain (PR8) was a gift from the National Institute for Viral Disease Control and Prevention, Chinese Center for Disease Control and Prevention.

The SPF C57BL/6 (6 weeks) mice were bought from Vital River (Certificate No. SCXK (Beijing) 2021-0006, China). All animal experiments were approved by the Welfare and Ethical Inspection at the Animal Experiment Center of the Chinese Center for Disease Control and Prevention (IACUC Issue No. 2024-034).

### 2.2. 16S rRNA Analysis

TIANamp Bacteria DNA Kit was used to extract *B. dorei*’s DNA (TIANGEN, Beijing, China), and a spectrophotometer was used to determine the concentration and purity of DNA products. The verification of culture identities was achieved through sequencing the 16S rRNA gene, employing universal bacterial primers. The acquired genomic sequences of target strains were subjected to nucleotide BLAST analysis through the NCBI platform (https://www.ncbi.nlm.nih.gov/ accessed on 26 February 2025) to obtain the 16S rRNA sequences of similar organisms. Phylogenetic analysis was subsequently performed using MEGA 12 software with the neighbor-joining algorithm to construct the evolutionary tree of *B. dorei*.

### 2.3. Genome Sequencing and Annotation

The draft genome sequencing of the strain *B. dorei* was performed using the illumina HiSeq TM2000/MiSeq. Quality control of raw sequencing data was conducted using fastp [13] with default parameters to eliminate adapter sequences and low-quality reads, yielding high-quality clean reads. Subsequently, these high-quality clean reads underwent de novo genome assembly using three independent assemblers: SOAP denovo [14], SPAdes [15], and ABySS [16], each configured with their respective optimal parameters. The resulting contigs from each assembler were then integrated and reconciled using CISA [17]. Finally, GapCloser was utilized for scaffolding optimization and gap closure to produce a complete genome assembly. GeneMarkS [18] was used to encode new sequencing of the genome of the gene prediction. Kyoto Encyclopedia of Genes and Genomes (KEGG) [19], Gene Ontology (GO) [20], carbohydrate-active enzymes (CAZy) [21] and Swiss-Prot [22] were utilized.

### 2.4. Bacteriocin and Secondary Metabolite

Bacteriocin identification and bacterial secondary metabolite profiling were, respectively, performed using BAGEL 4 and AntiSMASH bacterial version [23].

### 2.5. Tolerance to Simulation of Gastrointestinal Digestion

According to the Chinese pharmacopeia, artificial simulated gastric juice was prepared by dissolving 10 g of pepsin in a solution containing 16 mL of dilute hydrochloric acid and 950 mL of deionized water. The pH was adjusted to 3.0, and the volume was brought to 1 L with deionized water. The solution was then filtered through a 0.22 μm sterile filter. Simulated intestinal fluid was prepared by dissolving 3.4 g of KH_2_PO_4_ and 5 g of trypsin in 450 mL of deionized water. The pH was adjusted to 8.0, and the volume was brought to 500 mL with deionized water. The solution was then sterilized by filtration through a sterile filter. *B. dorei* was anaerobically cultured on plates for 24 h, followed by being harvested and resuspended in equal volumes of simulated gastric juice or intestinal fluids, respectively. After anaerobic incubation at 37 °C for 0, 30, 60, 120, and 180 min, bacterial samples were collected, serially diluted, and subjected to viable cell counting [24].

*B. dorei* was suspended in anaerobic basal broth medium containing varying concentrations of bile salts (0%, 2%, 3%, 4%, and 5% (*w*/*v*)) and incubated anaerobically at 37 °C for 24 h. Following incubation, bacterial solutions were serially diluted, and viable bacterial counts were determined [25].

### 2.6. Cell Adhesion Ability

Self-aggregation and hydrophobicity assays [26] were conducted to assess the adhesion capacity of *B. dorei*. Overnight cultures of *B. dorei*, *S. epidermidis*, and *L. plantarum* at 37 °C were harvested, resuspended in PBS buffer, and adjusted to an OD600 of 0.8 (A_0_). Aliquots of 4 mL bacterial suspension were incubated at 37 °C (n = 3 per group). After 24 h, the absorbance of the upper phase was measured at 600 nm (A_1_). The self-aggregation ability was calculated using the formula: A(%) = (1 − A_1_/A_0_) × 100.

Overnight cultures of *B. dorei, S. epidermidis*, and *L. plantarum* at 37 °C were harvested, resuspended in PBS buffer, and adjusted to an OD600 of 0.8 (H_0_). To each 3 mL of bacterial suspension, 1 mL of chloroform was added, followed by vortex mixing. The mixtures were then incubated at 37 °C (n = 3 per group). After 1 h, the absorbance of the aqueous phase was measured at 600 nm (H_1_), and bacterial hydrophobicity was calculated using the following formula: H(%) = (1 − H_1_/H_0_) × 100.

### 2.7. DPPH Free Radical Scavenging Assay

Overnight cultures of *B. dorei* were filtered to remove bacterial cells, and the remaining bacterial cells were adjusted to concentrations of 10^7^, 10^8^, and 10^9^ CFU/mL. A 0.4 mmol/L DPPH (Solarbio, Beijing, China) solution was prepared in absolute ethanol and combined with the bacterial solution/suspensions as per the experimental design. The mixtures were incubated at 37 °C in the dark for 30 min, and the absorbance was measured at 517 nm [27].

For the experimental setup, three groups were established. The experimental group (D_s_) was formed by mixing 2 mL of the bacterial solution or supernatant with 2 mL of the DPPH solution. The control group (D_c_) was created by combining 2 mL of the bacterial solution or supernatant with 2 mL of the anhydrous ethanol solution. The blank group (D_b_) was prepared by adding 2 mL of deionized water to 2 mL of the DPPH solution. The scavenging rate of DPPH was calculated using the formula: DPPH (%) = [1 − (D_s_ − D_c_)/D_b_] × 100.

### 2.8. Analysis of Antibacterial Activity

According to Drumond et al. [28], for experimental methods *B. dorei* was inoculated into liquid medium at 2% and cultured overnight. The culture was centrifuged at 4000 rpm for 10 min, and the supernatant was collected and sterilized by filtration. Pathogenic bacteria, including *E. coli* (EHEC), *S. typhimurium*, *S. aureus*, and *E. faecalis*, were suspended and adjusted to a McFarland standard of 0.5. Equal volumes of the pathogenic bacterial suspensions were mixed with either the *B. dorei* culture supernatant or blank medium and incubated at 37 °C for 24 h. After incubation, bacterial solutions were serially diluted, and viable bacterial counts were determined.

### 2.9. Analysis of Bile Salt Hydrolase Activity

Bile salt plates were prepared following Haleem et al.’s method [29] by supplementing anaerobic broth medium with 7.5 g/L agar, 0.3% (*w*/*v*) bile salt (Thermo Scientific™ Oxoid, Basingstoke, UK), and 0.375 g/L CaCl_2_. Sterile 6 mm filter paper disks were placed on the plates and inoculated with 10 μL of either sterile medium (control), *B. dorei*, or *L. plantarum* suspensions. Following 48–72 h incubation at 37 °C, precipitation ring formation was evaluated.

### 2.10. Hemolytic Activity

Filter paper disks were placed on Columbia blood agar and BHI agar (with 5% sheep blood) (Thermo Scientific™ Oxoid, Basingstoke, UK). Bacterial suspensions of *B. dorei, S. epidermidis*, and *L. plantarum* were prepared and adjusted to OD600 = 1.0, and 10 μL bacterial suspensions were inoculated onto the paper disks. After 48 h incubation at 37 °C, hemolytic activity was assessed by observing red blood cell lysis around the colonies.

### 2.11. Toxicity Experiment

To evaluate the safety of *B. dorei* in vivo, toxicity experiments were conducted. The experimental mice were numbered and then randomly assigned to three experimental groups (n = 5) using a random number generator. Each mouse was given either 200 μL 5 × 10^9^ CFU/d *B. dorei*, 5 × 10^10^ CFU/d *B. dorei*, or PBS by oral gavage for 14 days. At predetermined time points each day, the body weight was meticulously documented. After a 14-day observation period, the mice were euthanized, and their thymus and spleen weights were accurately recorded. The heart, liver, spleen, and colon were harvested for detailed histopathological examination. The changes in body weight, growth, immune organ index, and major organ pathology were compared with those of normal mice to evaluate whether the mice treated with *B. dorei* exhibited abnormal reactions.

### 2.12. Metabolomic Analysis

*B dorei* was grown anaerobically in a liquid medium for 24 h and then centrifuged to collect the supernatant for untargeted metabolomics. In brief, LC-MS/MS analysis was performed using a Thermo UHPLC-Q Exactive HF-X system. Raw data were processed with Progenesis QI 3.0 software for baseline filtering, peak identification, integration, retention time correction, and alignment. The resulting data matrix, which included retention time, mass-to-charge ratio, peak intensity, and MS/MS spectra, was annotated against the HMDB metabolomic database for metabolite identification. This data matrix was then uploaded to the Majorbio cloud platform (https://cloud.majorbio.com, accessed in 26 February 2025) for further analysis.

During pre-processing, retain at least 80% of metabolic features in any sample set. Estimate the minimum value for samples with metabolite levels below the quantification limit and normalize metabolic signatures to the sum. Apply the sum normalization method to mass spectrometry peak intensities to reduce errors from sample prep and instrument instability. Exclude QC sample variables with RSD > 30% and perform log10 transformation to obtain the final data matrix for further analysis.

### 2.13. RNA-Seq Quantification and RT-PCR

Total RNA was isolated from tissue homogenate by Trizol kit (TIANGEN, Beijing, China). The RNA samples extracted from BMDCs were utilized for the preparation of mRNA libraries and subsequent sequencing conducted by BGI (Wuhan, China). The data were processed and analyzed on the BGI Dr. Tom network platform, accessible at http://report.bgi.com (accessed in 26 September 2024). Converting the isolated tissues RNA into complementary DNA (cDNA) using reverse transcriptase (TIANGEN, Beijing, China) and specific primers (Table S1). The SYBR Green qPCR Master Mix (TIANGEN, Beijing, China) was used for Real-Time PCR amplification. Calculating the relative expression levels of the target gene was converted by normalizing the Ct values to the reference gene using the 2^−ΔΔCt^ method.

### 2.14. ELISA

The concentrations of cytokines in cultured cell supernatant was detected using the ELISA kit (Invitrogen, Waltham, MA, USA).

### 2.15. Cell Culture and Stimulation

Bone marrow cells were collected from C57BL/6J mice and cultured in RPMI 1640 medium supplemented with 20 ng/mL GM-CSF (R&D Systems, Minneapolis, MN, USA) and 20 ng/mL IL-4 (PeproTech, Waltham, MA, USA) for 6–7 days to obtain bone marrow-derived dendritic cells (BMDCs), or cultured in RPMI 1640 medium containing 20 ng/mL macrophage colony-stimulating factor (M-CSF, R&D Systems, Minneapolis, MN, USA) to obtain bone marrow-derived macrophage (BMDMs), following these described protocol [30,31]. MLE12 cells (ATCC CRL-2110) were maintained in DMEM medium (supplemented with 10% fetal bovine serum) (Gibco, Waltham, MA, USA). For the antiviral assay, BMDCs and BMDMs stimulated with *B. dorei* (MOI of 10), LPS (1 μg/mL, positive group) (Sigma-aldrich St. Louis, MO, USA), or PBS (control group) for 24 h were collected for IFN-β detection. Subsequently, BMDMs were infected with PR8 (MOI of 0.5) for 24 h. Simultaneously, MLE12 cells were pretreated with the culture supernatants of BMDCs for 6 h, followed by further infection with PR8 (MOI of 0.5) for 24 h. The viral load was detected by RT-PCR.

### 2.16. Statistical Analysis

All statistical analyses were conducted in GraphPad Prism 8 with appropriate methodological selection: Two-sample comparisons employed Student’s *t*-test, whereas multi-group datasets underwent one-way ANOVA verification. Experimental data, collected from a minimum of three biological replicates, are expressed as mean values with standard deviation (SD). Results were considered statistically significant at *p* < 0.05.

## 3. Results

### 3.1. Genetic Characteristics of B. dorei Strain

The complete genome sequence of this strain comprises 7,588,798 base pairs (bp) with a GC content of 40.81%. Genome prediction revealed 6783 protein-coding genes, representing 89.47% of the total genome sequence, with a total coding sequence length of 6,789,711 bp and an average gene length of 1001 bp. Additionally, 146 non-coding RNAs were identified, including 124 tRNA genes, 14 rRNA genes, and 8 sRNA genes (Table 1).

**Figure 1:**
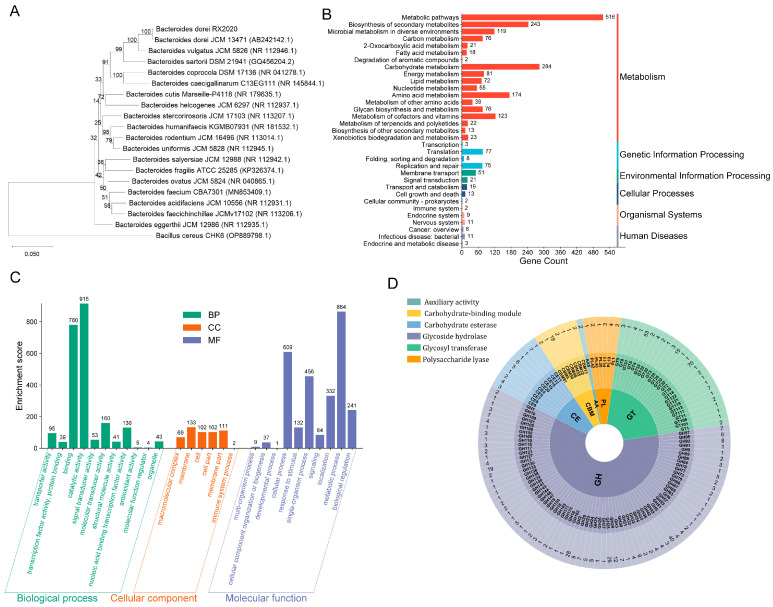
The gene function annotations of the *B. dorei*. (**A**) A phylogenetic tree constructed using a 16S rRNA sequence (neighbor-joining method). (**B**) KEGG database annotation results. (**C**) GO database annotation results. (**D**) Classification of the predicted CAZy from *B. dorei*.

To elucidate the phylogenetic characteristics of the isolated strain, 16S rRNA gene sequencing was performed. The obtained sequence was compared with homologous *Bacteroides* 16S sequences retrieved from the NCBI database using MUSCLE for multiple sequence alignment. Phylogenetic analysis was conducted using MEGA software to construct a neighbor-joining tree, which demonstrated that the isolated strain formed a distinct cluster with *B. dorei* JCM 13471, supported by 100% bootstrap value and short branch length, indicating their highest similarity. Furthermore, whole-genome sequence-based digital DNA–DNA hybridization (dDDH) and average nucleotide identity (ANI) analyses were performed between the isolated strain and *B. dorei* JCM 13471. The results revealed 98.5% dDDH and 100% ANI values between the type strain *B. dorei* JCM 13,471 and the isolated strain, confirming the taxonomic position of the target strain within the *B. dorei* species (designated as *B. dorei* RX2020) (Figure 1A).

KEGG annotation results showed that there were 1957 metabolism-related genes, mainly carbohydrate metabolism, biosynthesis of secondary metabolites, and amino acid metabolism (Figure 1B), indicating that *B. dorei* may play an important role in nutrient processing and metabolic interactions within the gut ecosystem, potentially contributing to host energy harvest and metabolic homeostasis. GO analysis of *B. dorei* revealed predominant associations with biological processes, particularly catalytic activities, molecular binding, and signal transduction mechanisms. Cellular component annotations highlighted membrane-related structures and cellular architecture, with a minor subset associated with immune system functions. Molecular function categorization demonstrated fundamental cellular operations, including localization and metabolic regulation (Figure 1C). CAZy database annotation of the *B. dorei* genome identified 597 CAZy genes, predominantly encoding 318 glycoside hydrolases and 181 glycosyltransferases. This extensive CAZy repertoire indicates robust carbohydrate metabolic capabilities, facilitating *B. dorei’*s intestinal colonization and potentially contributing to gut microbiota homeostasis (Figure 1D). These results show that *B. dorei* may have notable catalytic activity, binding abilities, and signal transduction, underscoring its metabolic efficiency and genetic potential. Moreover, the enrichment patterns suggest a genomic specialization in metabolic coordination and cellular maintenance, indicative of its adaptation to specific ecological niches.

### 3.2. Probiotic Properties of B. dorei Strains

**Figure 2:**
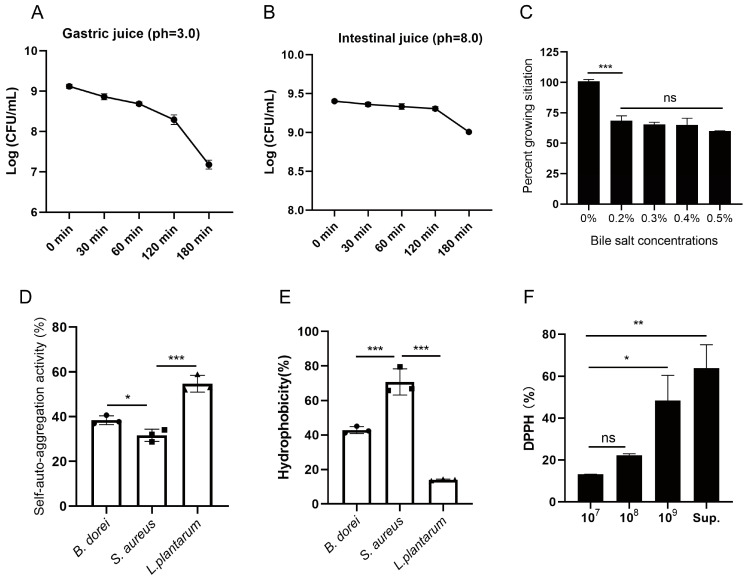
Evaluation of probiotic characteristics of *B. dorei*. (**A**) Evaluation of tolerance to artificial gastric juices. (**B**) Evaluation of tolerance to artificial intestinal juice. (**C**) Percentage of survival at different bile salt concentrations; (**D**,**E**) Auto-aggregation and hydrophobicity activity of *B. dorei*; (**F**) DPPH radical scavenging assay (Sup: the cell-free culture supernatant of *B. dorei*). The statistical analyses were assessed using Student’s *t*-test and one-way ANOVA. Statistical significance was defined as * *p* < 0.05, ** *p* < 0.01, and *** *p* < 0.001.

To assess gastrointestinal tract tolerance, *B. dorei* was subjected to simulated gastric juice (pH 3.0), intestinal fluid (pH 8.0) and bile salt medium. The strain could maintain 10^7^ CFU/mL after 180 min of exposure to simulated gastric juice. Intestinal fluid tolerance was more pronounced compared with gastric juice, with 10^9^ CFU/mL surviving after 180 min. Furthermore, *B. dorei* exhibited substantial bile salt resistance, showing 60% viability after 24 h exposure to 0.5% (*w*/*v*) bile salts (Figure 2A–C). The adhesion properties of *B. dorei* were comparable to those of *L. plantarum* and *S. aureus* (Figure 2D,E). The self-aggregation activity and hydrophobicity of *B. dorei* were intermediate (38.5% and 42.9%, respectively), while *L. plantarum* had the highest self-aggregation activity (54.8%) and *S. aureus* had the highest hydrophobicity (70.7%). To evaluate the antioxidant potential of *B. dorei*, we assessed the DPPH radical scavenging activity of both bacterial cells and cell-free culture supernatants. The results showed that *B. dorei* culture supernatant had antioxidant activity (63.8%) (Figure 2F), suggesting that extracellular metabolites may play a crucial role in the observed antioxidant activity.

Genome annotation of *B. dorei* via Swiss-Prot revealed multiple stress response genes (Table 2), including heat/cold shock proteins (HslO, Hsp18; CspLA, CspG) safeguarding intracellular proteins and aiding thermal adaptation. Molecular chaperones (ClpB, DnaJ/K, GroES/EL) ensured protein homeostasis. ClpP protease enhanced acid resistance by degrading denatured proteins, while ATP synthase sustained energy supply. Osmotic regulators (OpuAA, GbuA-C) and Na+/H+ exchangers (NhaA/P2/S4) stabilized pH/osmolarity. The thioredoxin system (TrxA/2/B, NTRC) and NADH peroxidase (Npr) neutralized oxidative damage, supporting redox balance and DNA repair [27,32,33]. Meanwhile, multiple cell surface adhesion-related genes were predicted in *B. dorei* (Table 3) that mediate binding to intestinal epithelial receptors, promoting colonization. Key genes include tuf (elongation factor Tu), malp (maltose phosphorylase), lspA (lipoprotein peptidase), gpac (glyceraldehyde-3P dehydrogenase), and tpiA (triose phosphate isomerase), with chaperones GroEL/ES enhancing adhesion efficiency [26,34]. Of course, the stress response genes and their corresponding functions need to be verified by subsequent experiments. These findings suggested that *B. dorei* exhibits probiotic characteristics to promote gastric and intestinal fluid tolerance, antioxidant capacity, and the ability to survive in the human gastrointestinal environment, thereby supporting intestinal health and homeostasis.

### 3.3. Antibacterial Properties

Foodborne illnesses pose a significant threat to global health through rising rates of illness and death. Bacterial foodborne pathogens primarily infect hosts through gastrointestinal mucosa invasion, with *Salmonella*, *Campylobacter jejuni,* and *Listeria monocytogenes* being common examples. Other pathogens like *E. coli* (EHEC), *S. aureus*, and *Clostridium botulinum* employ toxin-mediated mechanisms. Beneficial gut microbiota possessing antimicrobial properties play a vital role in preserving microbial equilibrium and overall health [35,36]. Our study demonstrated that *B. dorei* metabolites possessed antibacterial properties as they significantly inhibited the growth of foodborne pathogens, including *E. coli* (EHEC), *S. aureus*, and *S. typhimurium*. In contrast, these metabolites showed no inhibitory effect on the enteric symbiotic bacterium *E. faecalis*, and they even slightly promoted its growth (Table 4).

**Figure 3:**
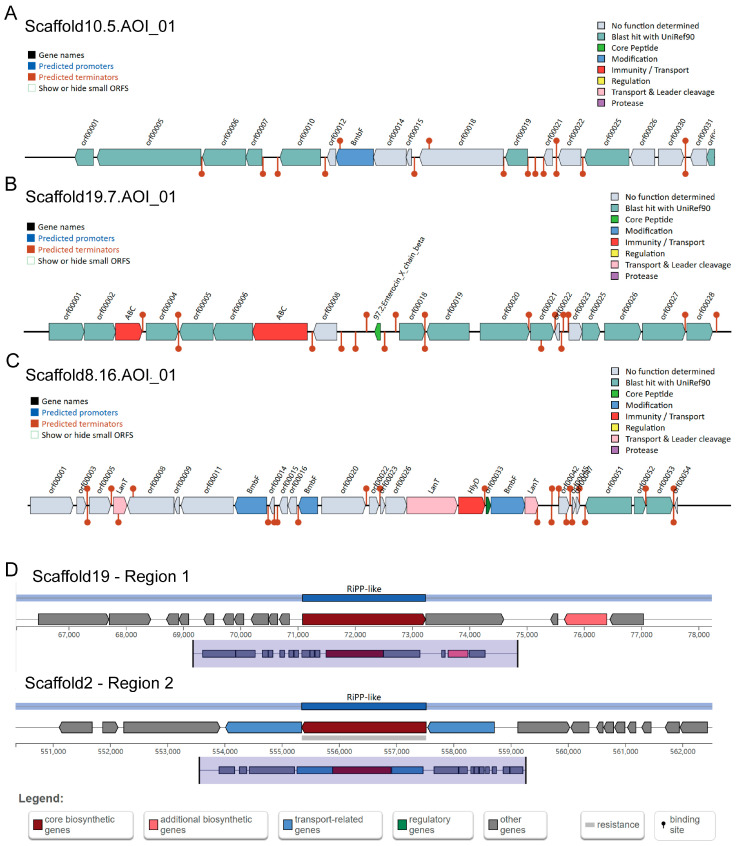
Bacteriocin gene clusters of isolated *B. dorei*. (**A**–**C**) Bacteriocin gene clusters of isolated *B. dorei* strain identified using BAGEL 4; (**D**) Bacteriocin gene clusters of isolated *B. dorei* strain identified using antiSMASH.

Genome-wide analysis of *B. dorei* using BAGEL 4 identified four bacteriocin-coding genes, including two Sactipeptide (Figure 3A), Enterocin X (Xbeta) (Figure 3B), and Bacteriocin_IIc (Figure 3C), which were further confirmed by NCBI BLAST. Enterocin X, a class IIb bacteriocin, shares homology with dipeptide antimicrobial peptides from *Enterococcus faecium* KU-B5 [37]. Bacteriocin_IIc, a class II bacteriocin, features immune genes, ABC transporters, and a double-glycine-type leader peptide, analogous to the colistin V secretion system in *Lactococcus lactis* [38]. AntiSMASH analysis revealed two secondary metabolite regions in *B. dorei*, each containing RiPP (ribosomally synthesized and post-translationally modified peptide) gene clusters (Figure 3D). Region 1 harbors five class IIb bacteriocins, an ABC transporter, and accessory proteins, while Region 2 includes a class IIb bacteriocin and an ABC transporter. RiPPs exhibit potent antibiotic activity by disrupting bacterial survival mechanisms, such as DNA replication, transcription, translation, and cell membrane integrity [39].

### 3.4. Bile Salt Hydrolase Activity

**Figure 4:**
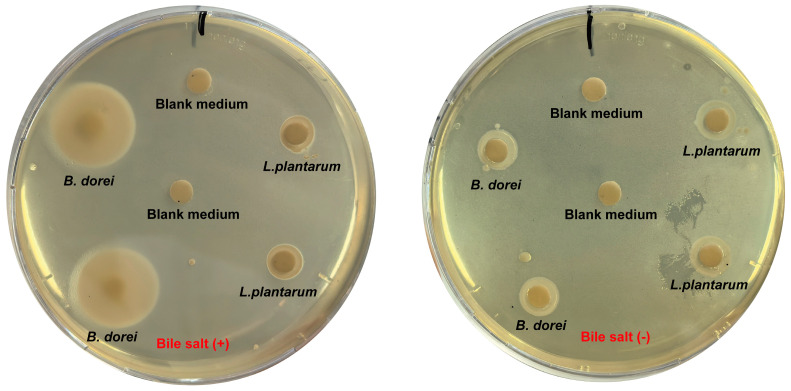
The BSH activity of *B. dorei*. The plate assay was conducted to determine BSH activity of *B. dorei* on Anaerobe Basal agar supplemented with 0.3% (*w*/*v*) bile salt and 0.37 g/L CaCl₂ ((**left**): media containing bile salt, (**right**): media without bile salt).

As a key bacterial hydrolase in the gut, BSH mediates the deconjugation process of bile acid conjugates, liberating unconjugated bile acids alongside amino acid byproducts. Free BAs facilitate cholesterol co-precipitation, thereby reducing cholesterol levels and enhancing lipid absorption and excretion [40]. Song et al. [41] classified BSH in the human gut microbiota into eight distinct types based on phylogenetic analysis. Sequence alignment revealed that *B. dorei* harbors two BSH types, BSH-T5 and BSH-T6 (Table 5). BSH activity of *B. dorei* was preliminarily confirmed using bile salt plates. Following bile salt deconjugation by *B. dorei*, free BAs reacted with calcium ions, forming a visible cloudy precipitate. In contrast, no cloudy precipitate was observed around *L. plantarum* colonies or in plates without bile salts (Figure 4).

### 3.5. Safety Evaluation

**Figure 5:**
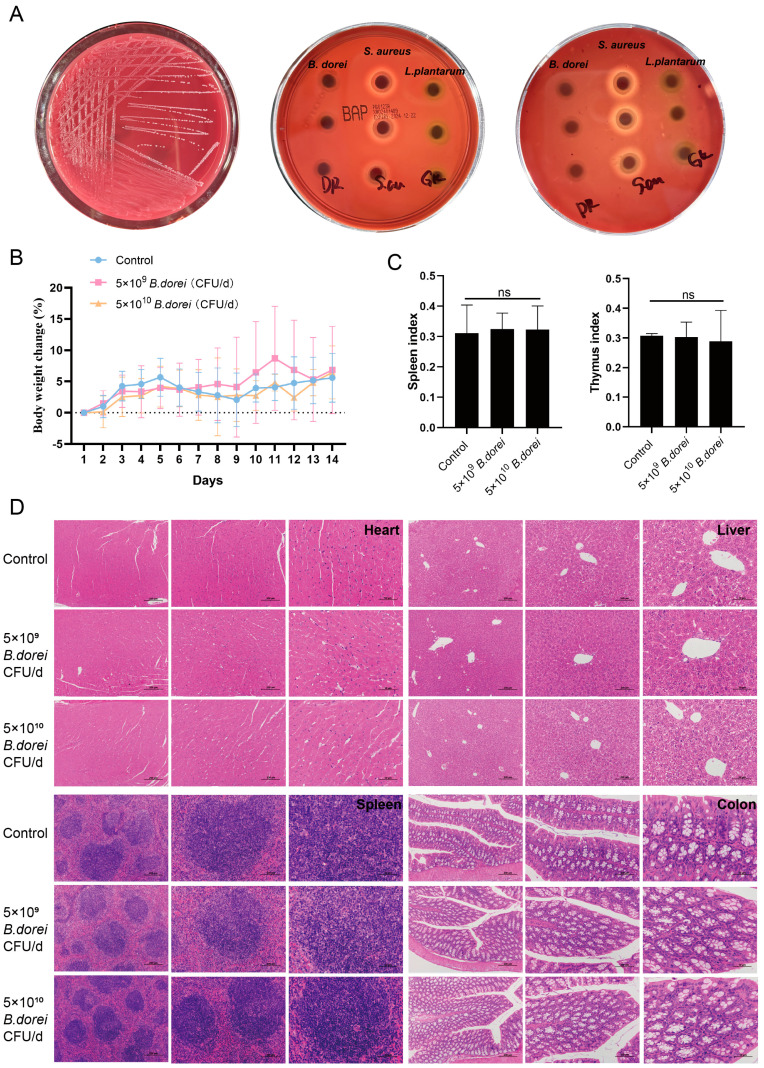
Safety assessment of *B. dorei*. (**A**) The hemolytic activities of the three strains were comparatively assessed by analyzing the hemolytic rings on both Columbia blood plates (middle) and BHI blood agar plates (right) (Left: *B. dorei* was incubated anaerobically on BHI blood plates for 48 h). SPF C57BL/6 normal mice were given either 5 × 10^9^ CFU/d, 5 × 10^10^ CFU/d *B. dorei*, or 0.2 mL/d PBS by oral gavage for 14 days. (**B**,**C**) Changes in the BW (%)/d and immune organ indices in the treatment and control groups. (**D**) Histopathological examination of heart, liver, spleen, and colon of mice in the control and treatment groups. The statistical analyses were assessed using one-way ANOVA. Statistical significance was defined as ^ns^ *p* > 0.5.

In our previous study, we predicted three virulence factor genes in the *B. dorei* genome, and none have been reported as pathogenetic genes. There were four antibiotic resistance genes, none of which were on the plasmid. Consistent with the prediction of antibiotic resistance genes, antibiotic susceptibility tests confirmed that *B. dorei* was resistant to cephalosporins and streptomycin, and sensitive to clarithromycin, clindamycin, tetracycline, penicillin, gentamicin, doxycycline, and ciprofloxacin [30]. *B. dorei* hemolysis test results are shown in Figure 5A. After 48 h of culture on Columbia blood plate and BHI plate (with 5% sheep blood), *B. dorei* colonies formed around the paper disk, with no hemolytic zone observed. In contrast, *S. aureus*, used as a positive control, exhibited a distinct β-hemolytic zone surrounding its colonies. There is no hemolysis during *B. dorei* culture, which may cause less risk of adverse reactions.

To evaluate the safety of *B dorei* in vivo, toxicity experiments were conducted. Each mouse was given either 5 × 10^9^ CFU/d *B. dorei*, 5 × 10^10^ CFU/d *B. dorei*, or PBS by oral gavage for 14 days. Neither death nor treatment-related toxicity occurred in mice. Body weight did not differ significantly among these groups (Figure 5B). There were also no significant differences in immune organ indices between groups either (Figure 5C). No histopathological injury to the heart, liver, spleen, and colon was observed in these groups (Figure 5D). Toxicology tests showed that a short period of oral administration of 10^9^–10^10^ *B. dorei* caused no adverse reactions and had no adverse effects on major organs.

### 3.6. Metabolites of B. dorei

**Figure 6:**
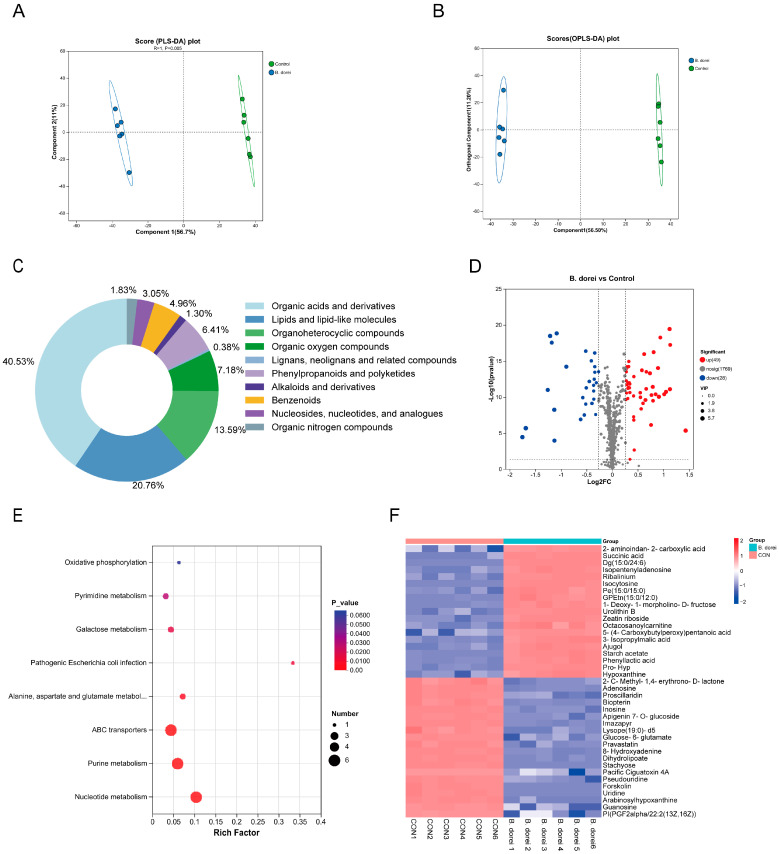
Metabolomics analysis of *B. dorei*’s culture supernatant. PLS-DA score plots (**A**), OPLS-DA score plots (**B**), species and contents of metabolite (**C**), the volcano map (**D**), KEGG pathway analysis (**E**), and heat map (**F**) of culture supernatant metabolites data from blank medium group and *B. dorei* group at 24 h.

To explore the metabolic activity of *B. dorei*, its culture supernatant was subjected to metabolomics. A partial least squares-discriminant analysis (PLS-DA) model demonstrated that metabolome samples from extracellular metabolites of *B. dorei* could be clearly separated from those treated with a culture medium (control) (Figure 6A). The Orthogonal Partial Least Squares-Discriminant Analysis (OPLS-DA) model demonstrated both stability and good predictive performance, as indicated by its R2Y (1) and Q2 (0.994) values close to 1 (Figure 6B). Analysis of Human Metabolism Database (HMDB) data showed that organic acids and derivatives accounted for the highest proportion (about 44.1%), indicating that bacterial metabolism is dominated by organic acid synthesis. Lipids and lipid-like molecules accounted for 22.6%, suggesting that membrane lipid metabolism is active (Figure 6C). Lipids not only have structural roles but also have potential effects on host health and physiology [42]. The volcano plot showed that there were 49 up-regulated metabolites and 28 down-regulated metabolites (fold change ≥1.2 or ≤0.8, *p* < 0.05, VIP ≥ 1) (Figure 6D). KEGG pathway analysis revealed significant enrichment in nucleotide metabolism, ABC transporters, purine and pyrimidine metabolism, and galactose metabolism (Figure 6E). The co-enrichment of these pathways suggests comprehensive metabolic reprogramming in the bacteria, characterized by enhanced energy production, active cellular proliferation, and adaptive responses to environmental challenges.

By utilizing heat maps for clustering different metabolites, we analyzed the 40 most significant metabolites (Figure 6F). We observed that the *B. dorei* group had upregulation of hypoxanthine and isocytosine, which are associated with nucleic acid metabolism and cellular proliferation, along with increased levels of DG, PE, and GPEtn, which are involved in lipid metabolism and membrane function. We also observed an elevation in levels of succinic acid and 3-isopropylmalic acid, the key intermediates in energy metabolism and carbon-nitrogen balance, which aligned with the genomic predictions. Moreover, we observed the upregulation of indole-3-acetic acid (IAA), phenyllactic acid, and the secondary BA deoxycholic acid (DCA). Although these metabolites did not rank among the most significantly altered, their changes were still statistically significant. *Bifidobacterium bifidum* effectively prevents hepatic steatosis and inflammation through the production of IAA, which alleviates dextran sulfate sodium (DSS)-induced colitis by promoting equol production from *Bifidobacterium pseudolongum* [43,44]. *Clostridium scindens* and/or its derived DCA can modulate pDC- and MyD88-dependent type IFN-I responses to restrict Chikungunya virus [45]. These results suggest that *B. dorei* may participate in the host immune response by regulating metabolism.

### 3.7. Immunomodulatory Activity of B. dorei

**Figure 7:**
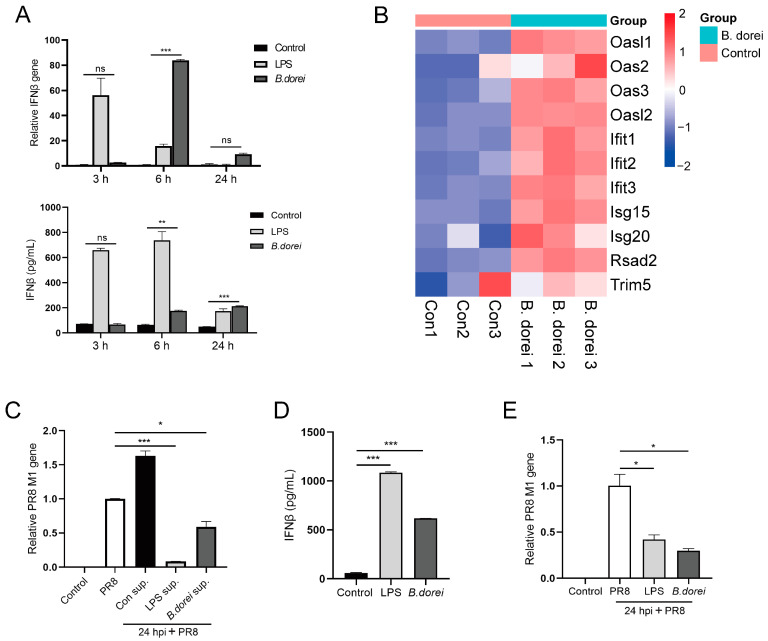
Immunomodulatory activity of *B. dorei*. (**A**) BMDCs were pretreated with *B. dorei* (MOI 10) for 3, 6, and 24 h. Relative IFNβ gene expression to β actin in BMDCs; IFNβ expression in culture supernatants from BMDCs. (**B**) Heat map of ISG and IFN gene expression. (**C**) Relative PR8 M1 gene expression to β actin in MLE12 cells. (**D**) IFNβ expression in culture supernatants from BMDMs. (**E**) Relative PR8 M1 gene expression to β actin in BMDMs. The statistical analyses were assessed using one-way ANOVA. Statistical significance was defined as * *p* < 0.05, ** *p* < 0.01, *** *p* < 0.001, and ^ns^ *p* > 0.5.

Based on the metabolomic results of *B. dorei*, we explored the immunomodulatory activity of *B. dorei* in vitro. As reported by Geva-Zatorsky N, there exists an association between *Bacteroides*, the number of pDCs, and the expression of IFN-I signature genes [46]. We first tested whether *B. dorei* could induce BMDCs to produce IFN-I in vitro. We then observed that *B. dorei*-treated BMDCs secreted IFN-β within 6 h (Figure 7A), and concurrently, *B. dorei* significantly upregulated the expression of ISG genes in BMDCs (Figure 7B). When this supernatant was used to treat MLE12 cells, a significant reduction in influenza virus replication was observed at 24 hpi (Figure 7C). Moreover, we also found that *B. dorei* was able to induce IFN-β production by macrophages, and similarly inhibited influenza virus replication (Figure 7D,E). These findings suggested that *B. dorei* was able to activate innate cells. *B. dorei* might enhance host defense against influenza viral infection through synergistic interactions with pulmonary epithelial cells, a mechanism potentially mediated by the augmentation of DC-mediated type I interferon signaling pathways via the regulation of BA metabolism.

## 4. Discussion

In recent years, the interplay between host and commensal bacteria in health and disease has gained significant attention as a key area of research. This study focused on the gut commensal bacterium *Bacteroides dorei* RX2020, evaluating its genomic characteristics, probiotic properties, metabolic functions, and immune activity. Our findings suggest that *B. dorei* possesses considerable potential in NGPs, providing critical insights into the physiological state of *B. dorei* and its potential mechanisms of interaction with the host.

Genomic functional analysis revealed that *B. dorei* was prominently involved in core metabolic pathways and the biosynthesis of secondary metabolites. These functional features were corroborated by subsequent metabolomic profiling. Notably, while secondary metabolites are non-essential growth components, they represent strain-specific bioactive compounds generated during bacterial proliferation. These molecules demonstrate multifaceted biological properties, conferring ecological competitive advantages with promising biotechnological applications [47,48]. In addition, *B. dorei* utilized a wealth of CAZy to generate its own carbon sources, while also contributing to the diversity of the gut microbiota and potentially influencing host health [49]. Differential analysis of disease and healthy CAZyme profiles confirmed underexpressed CAZyme markers in type 1 diabetes, colorectal cancer, and rheumatoid arthritis [50].

Robust assessment of probiotic viability within the gastrointestinal tract microenvironment is critical for therapeutic bioactivity realization. *B. dorei* demonstrated the ability to survive in simulated gastric fluid (pH 3) and intestinal fluid (pH 8). Although its survival rate significantly declined with extended exposure to a highly acidic environment, this may be attributed to its growth and resource utilization, which could have increased acidity beyond its tolerance threshold. This result is consistent with previous reports on properties of lactic acid bacteria [51]. *B. dorei* was also able to survive in 0.1–0.5% bile salts, which is an important indicator for the selection of probiotic candidates. Through BSH-mediated deconjugation of bile salts, probiotic bacteria attenuate bile toxicity, thereby enhancing their viability in the intestinal environment [52]. Highly hydrophobic bacterial strains exhibit enhanced adhesive properties toward mucosal surfaces, while their cellular autoaggregation capacity facilitates the formation of stable microbial communities within intestinal environments [53]. *B. dorei* possessed BSH genes and mucin-binding domains, which, in conjunction with its surface hydrophobicity and self-aggregating activity, facilitate its short-term colonization in the gut. Although it may not establish itself as a permanent resident in the gut like *Lactobacillus* and *Bifidobacterium* [54], regular consumption of these bacteria may still provide probiotic benefits without the need for colonization [55]. Follow-up toxicological assessments demonstrated that continuous oral dosing of *B. dorei* at elevated levels induced no detectable toxicity in mammalian models.

The gut microbiota, a complex community of microorganisms residing in the digestive tract, produces a variety of metabolites that play pivotal roles in modulating the host immune system. These metabolites, including short-chain fatty acids (SCFAs), tryptophan derivatives, and bile acids, act as critical messengers between microbial communities and immune cells, influencing both local and systemic immunity [56]. Gut microbiota-derived secondary bile acids modulate DCs and macrophages, while simultaneously exerting systemic anti-inflammatory effects on distant organs through the gut–X axis [57,58]. Gut microbial butyrate enhanced CD8+ T cell cytotoxicity via GPR109A/HOPX, thus, inhibiting gastric cancer carcinogenesis [59]. Our experimental validation of BSH activity in *B. dorei* coupled with DCA upregulation suggests that this strain may have the metabolic capacity to remodel BA profiles. The antiviral activity of *B. dorei* suggests its potential role in disease prevention and treatment, possibly mediated through modulation of tryptophan metabolism and BA metabolic pathways. Future research should focus on the antiviral and anti-inflammatory immunomodulatory effects of *B. dorei* and explore the immunomodulatory mechanisms of *B. dorei* in combination with models of viral infection, intestinal inflammation, and metabolic diseases. By correlating genomic potential with functional metabolism and safety parameters, this multidimensional strategy advances beyond conventional probiotic screening paradigms. The findings provide a template for rationally designing targeted microbial therapeutics while mitigating risks associated with clinical translation.

## 5. Conclusions

In conclusion, the gut commensal strain *B. dorei* RX2020 was able to resist gastrointestinal fluids, demonstrate auto-aggregation and hydrophobicity, combat foodborne pathogens, possess BSH and antioxidant activities, and exert immunomodulatory and metabolic effects during influenza virus infection. These findings complement prior research, underscoring the application potential of this strain as NPGs. Furthermore, while the strain has passed preliminary safety assessments, comprehensive investigations into its in vivo activity are imperative prior to advocating its incorporation into food additives.

## Figures and Tables

**Table 1:** *B. dorei* genomic characteristics.

Attribute	Values
Genome size (bp)	7,588,798
GC content (%)	40.81
Total genes	6783
Gene total length (bp)	6,789,711
Gene average length (bp)	1001
Gene length/Genome (%)	89.47
tRNA genes	124
rRNA genes	14
sRNA genes	8

**Table 2:** Stress response genes in *B. dorei*.

Gene Name	Annotation	Annotation No.
**Temperature stress**		
*groEL*	Chaperonin GroEL	A6KXA0; Q03VC3
*groES*	Co-chaperonin GroES	A6KXA1; Q03VC2
*hslO*	Heat shock protein 33 homolog	Q88Z30
*hsp18*	18 kDa heat shock protein	Q03928
*dnaJ*	Chaperone protein	O34136; Q5LED4
*dnaK*	Chaperone protein	A6L2 × 7; Q03WI2
*clpB*	Chaperone protein	Q8EU05; Q89YY3
*grpE*	Co-chaperone GrpE (heat shock protein)	Q03WI1; Q8A8C4
*cspLA*	Cold shock-like protein	P0A356
*cspG*	Cold shock-like protein	Q9S170
**PH stress**		
*atpA*	ATP synthase subunit alpha	A6L4M4
*atpB*	ATP synthase subunit a	B3QZE8
*atpC*	ATP synthase epsilon chain	Q04S19
*atpD*	ATP synthase subunit beta	A6L4L7
*atpE*	ATP synthase subunit c	A6L4M1
*atpF*	ATP synthase subunit b	A6L4M2
*atpG*	ATP synthase gamma chain	A6L4M5
*atpH*	ATP synthase subunit delta	A6L4M3
*clpA*	ATP-dependent Clp protease ATP-binding subunit	P0ABI1
*clpE*	ATP-dependent Clp protease ATP-binding subunit	Q9S5Z2
*clpX*	ATP-dependent Clp protease ATP-binding subunit	Q03W09; Q5L8L7
*clpP*	ATP-dependent Clp protease proteolytic subunit	B1MXG9; Q8A129
*clpS*	ATP-dependent Clp protease adapter protein	A3DER9
*nhaA*	Na(+)/H(+) antiporter	A6L743
*nhaP2*	K(+)/H(+) antiporter	A4XPR4
*nhaS4*	Na(+)/H(+) antiporter	P72973
**Osmotic stress**		
*opuAA*	Glycine betaine transport ATP-binding protein	Q9KIF7
*gbuA*	Glycine betaine/carnitine transport ATP-binding protein	Q9RR46
*gbuB*	Glycine betaine/carnitine transport permease protein	Q9RR45
*gbuC*	Glycine betaine/carnitine transport binding protein	Q9RR44
*proW*	Glycine betaine/proline betaine transport system permease protein	P14176
**Oxidative stress**		
*gpo*	Glutathione peroxidase	Q9CFV1
*gpx2*	Glutathione peroxidase-like peroxiredoxin 2	P38143
*nox*	NADH oxidase	P37061
*tpx*	Thiol peroxidase	Q71Z84; Q8KED5
*npr*	NADH peroxidase	P37062
*trxA*	Thioredoxin	O51088; P0A4L4
*trxB*	Thioredoxin reductase	O32823; P50971
*ntrc*	Thioredoxin reductase	Q70G58
*trx2*	Thioredoxin H2	Q38879

**Table 3:** The cell surface adhesion-related genes of *B. dorei*.

Gene Name	Annotation	Annotation No.
*malP*	Maltose phosphorylase	E6ENP7
*lspA*	Lipoprotein signal peptidase	B2RI39
*tuf*	Elongation factor Tu	A6KYK9; Q03YI2
*gpr*	L-glyceraldehyde 3-phosphate reductase	Q8 × 529
*gapc*	Glyceraldehyde-3-phosphate dehydrogenase, cytosolic	Q01558
*tpiA*	Triosephosphate isomerase	Q03SL6; A6KXL2
*groEL*	Chaperonin GroEL	A6KXA0; Q03VC3
*groES*	Co-chaperonin GroES	A6KXA1; Q03VC2

**Table 4:** Antibacterial activity of *B. dorei.*

Strains	Blank Medium	*B. dorei* Supernatant	
	Log CFU/mL	Log CFU/mL	Survival Rate
*E. coli* (EHEC)	8.05 ± 0.05	7.69 ± 0.12 ^a^	43.76%
*S. aureus*	7.75 ± 0.19	7.16 ± 0.35 ^b^	30.20%
*S. typhimurium*	8.15 ± 0.02	7.96 ± 0.04 ^a^	65.41%
*E. faecalis*	7.61 ± 0.16	7.63 ± 0.09	103.25%

Data are presented with mean ± S.D., statistical significance was defined as ^a^ *p* < 0.01, ^b^ *p* < 0.05.

**Table 5:** Sequence alignment of bile saline hydrolase gene.

Type	Genus	Description	Query	E Value	Per. Ident	Acc. Len
BSH-T0	*Enterococcus*	674_gene_2740	89%	6 × 10^−33^	28.75%	359
BSH-T1	*Eubacterium*	674_gene_2740	94%	1 × 10^−34^	30.51%	359
BSH-T2	*Streptococcus*	674_gene_2740	97%	1 × 10^−36^	30.03%	359
BSH-T3	*Lactobacillus*	674_gene_2740	98%	2 × 10^−33^	26.04%	359
BSH-T4	*Bifidobacterium*	674_gene_2740	92%	1 × 10^−38^	30.72%	359
BSH-T5	*Bacteroides*	674_gene_2740	94%	4 × 10^−153^	59.18%	359
BSH-T6	*Bacteroides*	651_gene_1492	97%	0	86.26%	352
BSH-T7	*Blautia*	No significant similarity found				

## Data Availability

RNAseq data generated during the current study are available in the NCBI SRA (SRA data: PRJNA1168481).

## Supplementary Materials

The following supporting information can be downloaded at: https://www.mdpi.com/article/10.3390/nu17061066/s1, Table S1: Primer sequences for qRT–PCR assay.
