# Supplementary material for: Genomic, Probiotic, and Functional Properties of Bacteroides dorei RX2020 Isolated from Gut Microbiota
Source: Nutrients. 2025 Mar 18;17(6):1066. doi: 10.3390/nu17061066 (PMC11944543; doi:10.3390/nu17061066)
Supplement: Supplementary file 1 [file nutrients-17-01066-s001.zip › nutrients-3529051-supplementary.pdf]

Supplementary Materials

Table S1: Primer sequences for qRT–PCR assay

Gene	Forward primer sequence (5’-3’)	Reverse primer sequence (5’-3’)
IFN-β	TCTGGAGCATCTCTTGGATGGCAA	TCCAGCTCCAAGAAAGGACGAACA
PR8 M1	AAGACCAATCCTGTCACCTCTGA	CAAAGCGTCTACGCTGCAGTCC
β-actin	CCTGGCACCCAGCACAAT	GCCGATCCACACGGAGTA
